# Transcriptomic-Based Identification of miR-125a Novel Targets in Human Hepatocarcinoma Cells

**DOI:** 10.3390/biom15010144

**Published:** 2025-01-18

**Authors:** Ilenia De Leo, Nicola Mosca, Mariaceleste Pezzullo, Danila Valletta, Francesco Manfrevola, Vincenza Grazia Mele, Rosanna Chianese, Aniello Russo, Nicoletta Potenza

**Affiliations:** 1 Department of Environmental, Biological and Pharmaceutical Sciences and Technologies, University of Campania “Luigi Vanvitelli”, 81100 Caserta, Italy; ilenia.deleo@unicampania.it (I.D.L.); nicola.mosca@unicampania.it (N.M.); mariaceleste.pezzullo@unicampania.it (M.P.); danila.valletta@unicampania.it (D.V.); aniello.russo@unicampania.it (A.R.); 2 Genomix4Life S.r.l., 84081 Baronissi, Italy; 3 Department of Experimental Medicine, University of Campania “L. Vanvitelli”, 80138 Naples, Italy; francesco.manfrevola@unicampania.it (F.M.); vincenzagrazia.mele@unicampania.it (V.G.M.); rosanna.chianese@unicampania.it (R.C.)

**Keywords:** RNAseq, miRNA target prediction, HCC, miR-125a, Lin-28

## Abstract

Hepatocellular carcinoma (HCC) is among the most aggressive and lethal human tumors. Many functional studies have demonstrated the role of non-coding RNAs (ncRNA), particularly microRNAs (miRNA), in the regulation of hepatocarcinogenesis driving pathways. MiR-125a-5p (miR-125a) has been consistently reported as an oncosuppressive miRNA, as demonstrated in vivo and in vitro. However, its HCC relevant targets and molecular mechanisms are still largely unknown. Here, a genome-wide perspective of the whole miR-125a targetome has been achieved. In particular, two different HCC cell lines were subjected to a miRNA boosting by mimic transfections, and consequently many genes were de-regulated, as observed by a transcriptomic approach. The merging of down-regulated genes with results from bioinformatic predictive tools yielded a number of candidate direct targets that were further experimentally validated by luciferase-based reporter assays. Different novel targets were found, in particular ARID3A, CCNJ, LIPA, NR6A1, and NUP210, oncogenes in various tumors and here also related to HCC through miR-125a regulation. The RNA interactions investigated in this work could pave the way to piece together the RNA regulatory networks governed by the miRNA impacting on hepatocarcinogenesis, and be exploited in the future for identifying novel biomarkers and therapeutic targets in HCC.

## 1. Introduction

Hepatocellular carcinoma (HCC), the predominant form of liver cancer, is one of the most common aggressive human tumors, ranking third for cancer-related death worldwide [1]. More than 80% of human HCCs arise from chronic liver disease as a consequence of viral hepatitis, alcohol abuse, exposure to carcinogenic agents, metabolic syndrome, and genetic diseases such as Wilson’s disease and hemochromatosis [2,3]. Various oncogenic or tumor suppressor proteins and molecular mechanisms contributing to the onset and progression of HCC have been so far characterized, such as the Ras mitogen-activated protein kinase (Ras/Raf/MAPK), the phosphatidylinositol 3-kinase (PI3K)/AKT/mammalian target of rapamycin (mTOR) and the Wnt/beta-catenin signaling pathways, the ubiquitin/proteasome degradation and the hedgehog signaling pathway [4].

More recently, a plethora of functional studies have demonstrated the role of non-coding RNAs (ncRNA), specifically microRNAs (miRNA), but also long non-coding RNAs (lncRNA) and circular RNAs (circRNA), in the regulation of hepatocarcinogenesis driving pathways [5]. In particular, miRNAs have been implicated from early to advanced stages in HCC, acting as either powerful oncogenes or tumor suppressors [6,7].

Mechanistically, miRNAs can affect the stability and translation of their target transcripts by directly and physically binding to complementary target sequences. In particular, a perfect match between the miRNA seed sequence (nucleotides 2–8) and target sequence is required, but mismatches are tolerated in the remaining part [8]. Therefore, one miRNA can have multiple transcript targets, and vice versa one transcript can have more than one miRNA binding site. Consequently, even one miRNA dysregulation can have a severe impact on gene expression at genome-wide level and on multiple pathways. In addition, miRNAs can also regulate gene expression through the so-called competing endogenous RNA networks (ceRNET), complex RNA regulatory networks wherein different RNA biotypes, coding and non-coding RNAs, compete with each other for binding to shared miRNAs, thus titrating miRNAs availability and reciprocally modulating their expression, even if they are functionally unrelated [9,10,11,12].

Many studies (by October 30th, 2024, 524 articles could be retrieved from PubMed searching by “miR-125a AND cancer”) focused their attention on hsa-miR-125a-5p (miR-125a), which is endowed with an oncosuppressive role in different tumor types, including HCC [13,14].

First, it is able to limit tumor growth in vivo [15,16]. It is down-regulated in HCC biopsies in comparison to adjacent non-tumoral tissues, as expected for a tumor suppressor, and is associated with patients’ poor survival [17,18]. It is involved in tumoral microenvironment setting, since its deficiency in exosomes derived from tumor-associated macrophages promotes the proliferation, stem cell properties, and metastatic capacity of HCC cells, whereas its re-expression suppresses the growth and sphere formation ability of liver cancer [19]. In different HCC cell lines, miR-125a is able to inhibit cancer hallmarks such as cell proliferation, migration and invasion, and is able to mediate the activity of Sorafenib, a drug representing the standard of care for advanced HCC [16,20,21]. Interestingly, it is involved in the hepatitis B virus infection, one of the causative factors of HCC [3]. With regard to molecular mechanisms, some miR-125a targets have been validated in HCC cells, such as matrix metalloproteinase-11 (MMP11), sirtuin-7 (SIRT-7), Zbtb7a, and the stemness factor Lin28b [16,21,22,23,24,25]. Intriguingly, Zbtb7a and Lin28b proteins are also able to transcriptionally and post-transcriptionally repress miR-125a, giving rise to a multifaceted liaison between miRNA biogenesis and its silencing activity [23,24,25]. In addition, miR-125a can interact with different lncRNAs, such as SPACA6P-AS, NEAT1, and CYTOR, building up ceRNETs where aberrant expression of any circuit component could derail the network with pathological consequences [26,27,28].

This work aims to gain a genome-wide perspective of the whole miR-125a targetome, so far mainly based on the prediction/validation of single targets, thus paving the way to piece together the RNA regulatory networks governed by the miRNA and impacting on hepatocarcinogenesis.

## 2. Materials and Methods

### 2.1. DNA Constructs

Luciferase reporter constructs were prepared as follows: the sequences potentially targeted by miR-125a were obtained through the chemical synthesis of complementary oligonucleotides (Invitrogen, Waltham, MA, USA) including upstream XhoI and EcoRV restriction sites and a downstream NotI site. Once annealed, the couple of oligonucleotides were ligated into XhoI and NotI restriction sites of the psiCHECK-2 vector (Promega, Madison, WI, USA). Digestions with *Eco*RV were then used to screen the recombinant clones. Control plasmids were obtained using the same approach, with the exception that the cloned couple of oligonucleotides represented the inverted target sequence. Sequencing was used to confirm the identity of all the constructs.

### 2.2. Cell Cultures, Transfections, and Luciferase Assays

Human liver cancer cell lines HepG2 and HuH-7 (ATCC, Manassas, VA, USA) were cultured in RPMI 1640 and DMEM, respectively, containing 10% fetal bovine serum, 2 mM L-glutamine, 50 U/mL penicillin, and 100 μg/mL streptomycin. The day before transfection, cells were trypsinized and seeded in medium without antibiotics in 12-well plates. Transfections were then performed with cells at 80–90% confluence by using 3 μL of Lipofectamine2000 (Thermo Fisher Scientific, Waltham, MA, USA) for 1 μg of nucleic acids, as described by the manufacturer. In particular, the miR-125a mimic or its control (Ctrl-miRNA) with unrelated sequence (all from Dharmacon, Lafayette, CO, USA) were transfected at 50 nM. 0.05 μg or 0.2 μg of reporter constructs were used for transfecting HuH-7 or HepG2 cells, respectively. The transfection mix was replaced with complete medium after 6 h, and the analyses were performed 48 h after transfection. Luciferase assays were performed using the Dual-Luciferase Reporter Assay System (Promega, Madison, WI, USA) according to the manufacturer’s protocol.

### 2.3. RNA Purification and Quantitative PCR Analyses

Total RNA was extracted from cell cultures using the miRNeasy mini kit (Qiagen, Hilden, Germany). The yield and quality of RNA were evaluated using the NanoDrop 2000c spectrophotometer (Thermo Fisher Scientific, Waltham, MA, USA), Qubit 4 Fluorometer (Invitrogen, Waltham, MA, USA), and TapeStation 4200 (Agilent Technologies, Santa Clara, CA, USA).

In order to check the increased level of hsa-miR-125a-5p after transfections, it was quantified along with RNU6B (reference transcript) using RT-qPCR with TaqMan miRNA assays (Applied Biosystems, Waltham, MA, USA) according to the manufacturer’s protocol.

For quantification of the miR-125a target transcripts, total RNA was retrotranscribed using the SensiFAST cDNA Synthesis kit (Bioline, London, UK). Then, standard SYBR Green Real-time qPCR assays were performed with the following primers:

ARID3A, 5′-ACCACGGCGACTGGACTTA-3′ and 5′-CACAGGTGTCCCTCGCTTC-3′ [29];

CCNJ, 5′-CTGGCCGCCGATATTCACC-3′ and 5′-GGCAATCAAGTCAGCAAAATACC-3′ [30];

LIPA, 5′-ACAGATCCCTGAGCTGGCTA-3′ and 5′-AGGGCTAGTACAGAAGGCGA-3′;

NR6A1, 5′-CCCTCCGATGAAGAACTACACAGAT-3′ and 5′-GCATACTCCTCGTTGCTGACCT-3′ [31];

NUP210, 5′-GGTCATGATCATAGCCTACCACA-3′ and 5′-GCATTGGGAGATGTGGGTGA-3′ [32];

GAPDH (reference transcript), 5′-GAAGGTGAAGGTCGGAGTC-3′ and 5′-GAAGATGGTGATGGGATTT-3′.

The expression levels of miRNA and other transcripts were normalized to their respective reference transcripts and to those of the control conditions by the 2−ΔΔCtmethod [33].

### 2.4. RNA Sequencing and Data Analyses

Based on the best increased expression of miR-125a expression after miRNA mimic transfection, as evaluated according to the procedure of the above paragraph, three miR-125a mimic transfected HuH-7 cell samples and the related three Ctrl-miRNA mimic transfected cell samples were selected for RNAseq experiments; the same was conducted for HepG2 cells. Indexed libraries were prepared from 100 ng of each purified RNA sample using the Illumina Stranded Total RNA Prep, Ligation with Ribo-Zero™ (Illumina, San Diego, CA, USA), according to the manufacturer’s instructions. Libraries were quantified with the TapeStation 4200 (Agilent Technologies, Santa Clara, CA, USA) and Qubit fluorometer (Invitrogen, Waltham, MA, USA). Libraries were pooled such that each index-tagged sample was present in equimolar amount. The pooled samples were subjected to cluster generation and sequenced using Illumina NextSeq 550 Dx System (Illumina) in a 2 × 101 paired-end format, with an average depth of 30 million reads/sample. The generated raw sequence files (.fastq files) underwent quality control analysis using FastQC (http://www.bioinformatics.babraham.ac.uk/projects/fastqc/, accessed on 7 March 2023). The bioinformatic tool cutadapt (version 2.5) [34] was used to remove the adapter sequences and the very short reads (reads length < 25) in order to profile coding and non-coding RNAs in all sequenced total RNA samples. Then, each sample was mapped on reference genome (https://www.gencodegenes.org/) using the bioinformatic tool STAR (version 2.7.5c) [35], with standard parameters for paired reads. The reference track was the assembly Human obtained from GenCode [HG38-Release37 (GRCh38.p13)] (https://www.gencodegenes.org). The quantification of transcripts expressed for each sequenced sample was performed using featureCount algorithm [36]. The Bioconductor package DESeq2 version number 1.34.0 [37] in R version number 24.04.2 was used to normalize the data using the median of ratios method, and to perform the differential expression analysis between the various experimental conditions. Genes were considered differentially expressed according to the following parameters: *p*-value cut-off: 0.05; fold change cut-off: ±1.3. Finally, we performed Euclidean distances (Heatmap Distances) and Principal component analysis (PCA) among all samples in each considered condition to evaluate the general similarity between the samples.

The enrichment of biological pathways supplied by Kyoto Encyclopedia of Genes and Genomes (KEGG) pathway annotation was performed by R-package PathFinder version number 2.4.1 (pathfindR) [38]. To control the false-positive rate in the results, a multiple test correction of enrichment *p*-values was performed on the functional annotation categories by selecting the Benjamini–Hochberg procedure; biological pathways with *p*-values < 0.05 were considered statistically significant.

miR-125a target prediction analyses were performed using miRWalk (http://mirwalk.umm.uni-heidelberg.de/, accessed on 3 May 2023) and RNAhybrid tools (http://bibiserv.cebitec.uni-bielefeld.de/rnahybrid, accessed on 9 May 2023).

## 3. Results

### 3.1. Rationale of the Study

**Figure 1:**
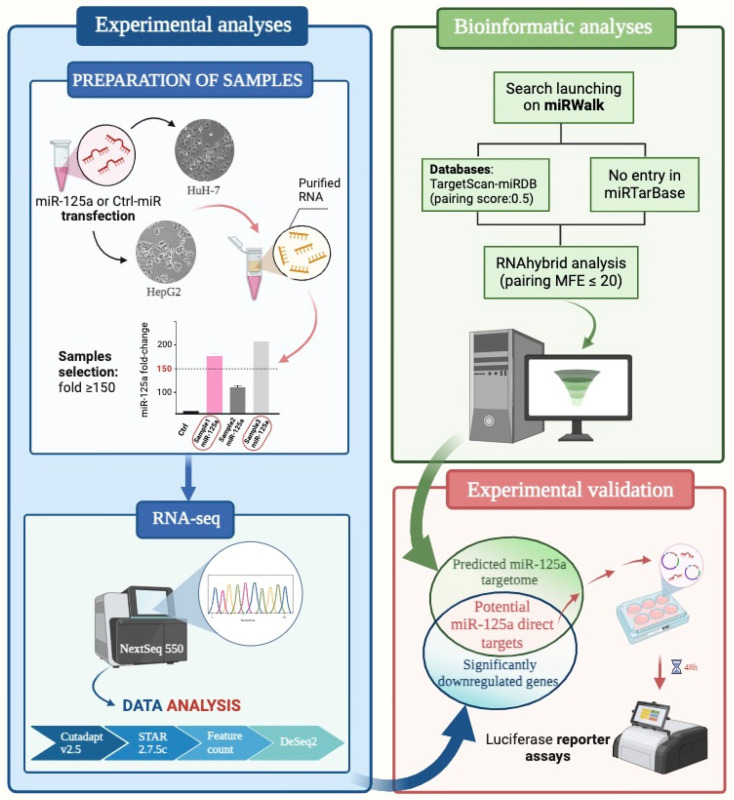
Workflow of the study. Figure created with BioRender.com.

In order to select novel direct targets of miR-125a, we set up a rigorous workflow based on the merging of experimental data and bioinformatic analyses. The starting point of experimental data was the boosting of intracellular level of miR-125a in order to observe possible change in gene expression (Figure 1, left panel): HuH-7 and HepG2 cells, representing a model for HCC, were both transfected with miR-125a mimic or the control miRNA molecule. After having evaluated the increased expression of the miRNA in comparison to the control (ranging from 150- to 200-fold), samples were subjected to RNAseq experiments. Result analyses led to lists of significantly up-regulated or down-regulated genes as a consequence of miR-125a increased level (Supplementary S1: sheet 1-DE_HepG2, sheet 2-DE_HuH-7). Taking into account the general mechanism of miRNA regulation, we focused our attention on the down-regulated genes. In parallel, the whole predicted targetome of miR-125a was obtained by launching a search on miRWalk platform (http://mirwalk.umm.uni-heidelberg.de/), using predictive bioinformatic tools TargetScan and miRDB, but excluding the validated targets as reported in miRTarBase (Figure 1, right panel). In detail, by selecting “miRTarBase”, or “TargetScan” and “miRDB”, and “5′ UTR”, or “3′ UTR” or “coding region” and selecting the lower score (0.5) for miRNA–mRNA pairings, six different target lists were retrieved containing all experimentally validated targets, or those predicted by both TargetScan and miRDB tools. By exporting the lists in Excel, a unique list of validated targets (Supplementary S1: sheet 3-miRTarBasevalidated targetome) and predicted non-redundant transcripts representing the whole targetome of miR-125a was produced (Supplementary S1: sheet 4-Predicted targetome). Finally, the miRNA–mRNA pairings were further analyzed using RNAhybrid (http://bibiserv.cebitec.uni-bielefeld.de/rnahybrid) to select those to be experimentally validated. The merging of the down-regulated gene lists as derived from RNAseq experiments and the others derived from bioinformatic prediction, gave us potential direct targets of the miRNA to experimentally validate (Supplementary S1: sheet 5-Merging HepG2, sheet 6-Merging HuH-7).

### 3.2. Transcriptomic Changes Induced by Increased miR-125a Levels

**Figure 2:**
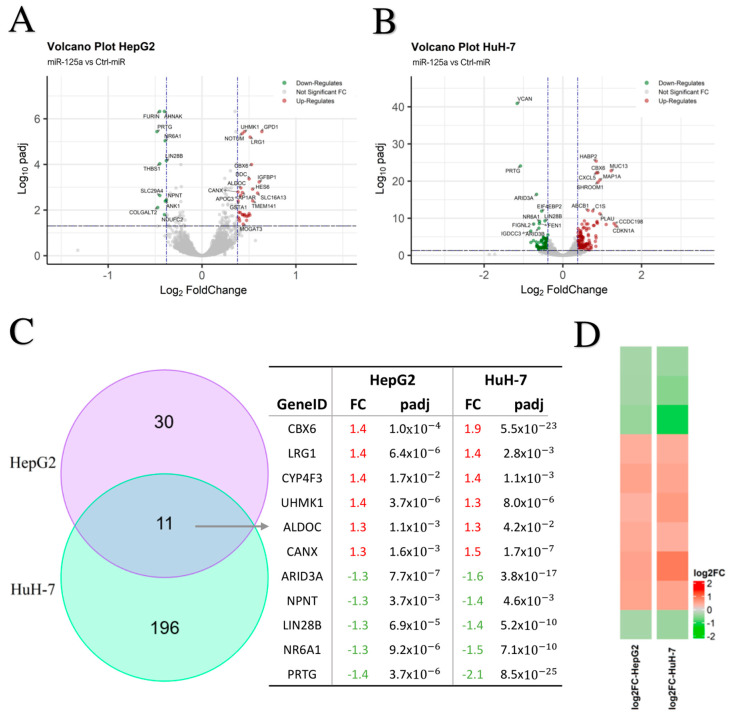
1.RNAseq results analyses. (**A**) Volcano plots of differentially expressed genes in miR-125a mimic transfected HepG2 cells and (**B**) HuH-7 cells, performed by RStudio 24.04.2 using Enhanced Volcano 1.24 package. Up-regulated genes are in red, down-regulated genes in green, and unchanged genes in gray (*p*-value cut-off: 0.05, fold change cut-off: ±1.3). (**C**) On the left, Venn Diagram of differentially expressed genes in miR-125a mimic transfected HepG2 cells (violet), HuH-7 cells (light blue) and both (in the intersection) in comparison to Ctrl-miR transfected cells, performed by RStudio using VennDiagram 1.7.3 package; on the right, table with genes differentially expressed in both cell lines (intersection of Venn diagram). (**D**) Heatmap of log2Fold change of common differentially expressed genes in HepG2 and HuH-7 samples, performed by RStudio. padj, *p*-value adjusted by selecting the Benjamini–Hochberg procedure.

HepG2 and HuH-7 showed up-regulated and down-regulated genes as a consequence of miR-125a increased level by mimic transfection, with some of them specifically de-regulated in a single cell line, and others commonly de-regulated (Figure 2). The up-regulated genes are probably indirectly linked to miR-125a function, e.g., the miRNA could target a transcription factor affecting the expression of a specific gene that consequently was up-regulated. Vice versa, the down-regulated genes could be specific and direct targets of the miRNA. Notably, among the down-regulated genes, as a consequence of miR-125a increased level, some have been consistently validated from different published works, such as Lin28 from nematodes to humans, thus increasing the confidence in the obtained results (Supplementary S1). Intriguingly, a lower number of genes appeared deregulated in HepG2 in comparison to HuH-7, probably because of already higher expression of the endogenous miR-125a in HepG2, as experimentally demonstrated elsewhere [26].

**Figure 3:**
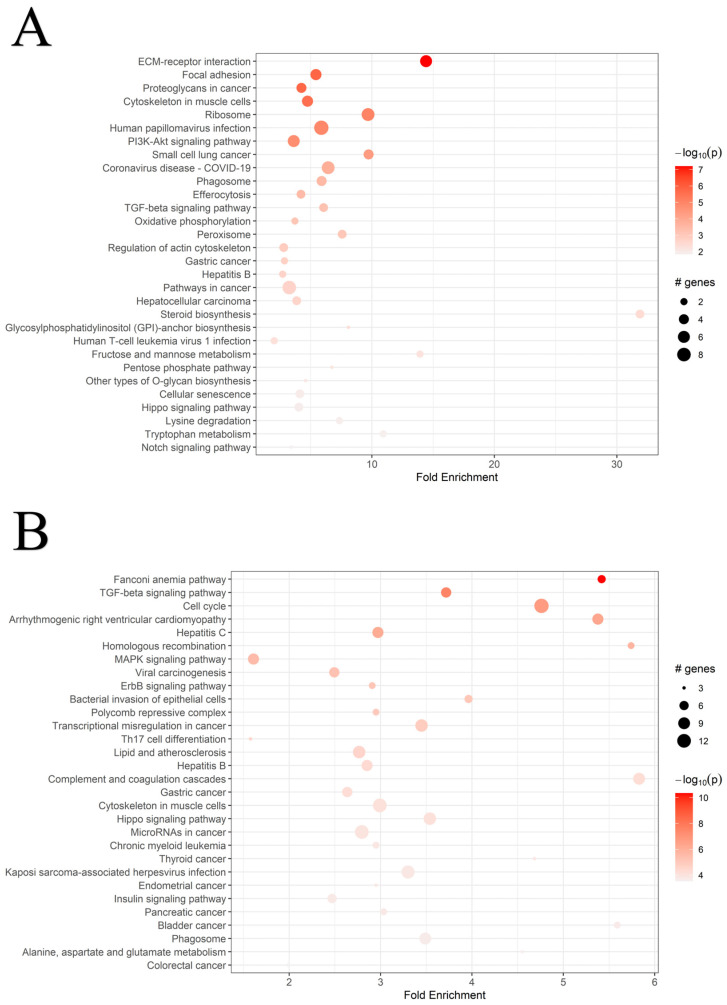
Pathway enrichment analyses of differentially expressed genes as a consequence of miR-125a increased level. Bubble Charts representation showing pathway enrichment of DE-genes for HepG2 (**A**) and HuH-7 (**B**) cells performed by R-package Pathfinder (PathfindR). Pathway and gene set annotations arise from Kyoto Encyclopedia of Genes and Genomes (KEGG). The *x*-axis corresponds to fold enrichment values, while the *y*-axis indicates the top 30 enriched terms (full lists in Supplementary S2); the bubble size indicates the number of significant genes in the given enriched term; color indicates the −log10 (lowest-*p*) value, wherein the more intense the color, the more enrichment statistical significance.

In order to gain some functional insights into gene lists generated from omics experiments, we also analyzed and identified statistically enriched biological pathways in DE-genes from both cell lines (Figure 3 and Supplementary S2). Interestingly, the most represented biological terms are cancer, including HCC, and related signaling pathways in both cell lines analyses. Hepatitis B virus infection also statistically emerged from the analyses, consistent with the demonstrated role of miR-125a in molecularly balancing the infection severity with the host survivor [39].

### 3.3. Identification of Potential miR-125a Targets and Experimental Validation

**Figure 4:**
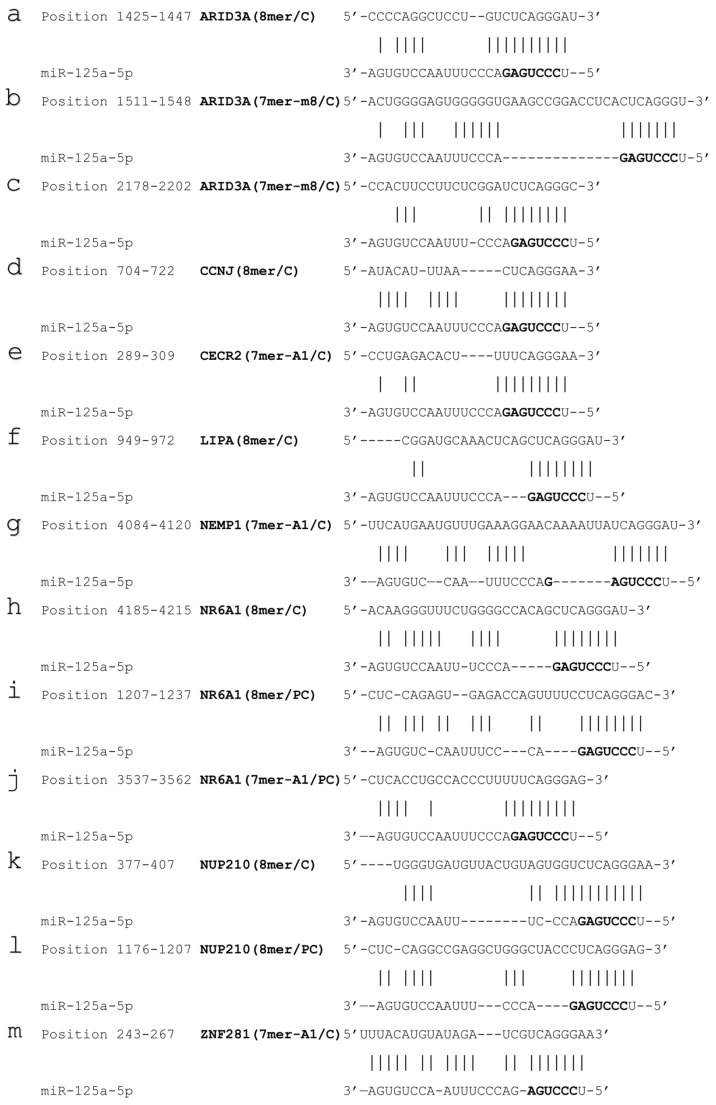
Pairings between miR-125a-5p sequence and the indicated targets. Specific predicted binding sites are indicated by the lowercase letters a to m. In addition to pairings with an exact match to positions 2–8 (in bold) of the mature miRNA followed by an “A” (8mer), two types of 7mer sites have also been included in the analysis, i.e., an exact match to positions 2–8 of the mature miRNA (7mer-m8), and an exact match to positions 2–7 of the mature miRNA followed by an “A” (7mer-A1), as defined by TargetScan; C or PC abbreviation indicates evolutionary “conserved” or “poor conserved” target sites.

In order to identify putative direct miR-125a targets, our attention was focused on down-regulated genes from RNAseq experiments, not yet validated (no entry in miRTarBase), but predicted by both TargetScan and miRDB, and further selecting those showing the perfect matching of the miRNA seed sequence also by RNAHybrid and with a minimal free energy < −20. The analyses yielded 13 potential pairings, with 3 different sites on ARID3A and NR6A1, 2 on NUP210, and one for all the other putative transcripts (Figure 4).

**Figure 5:**
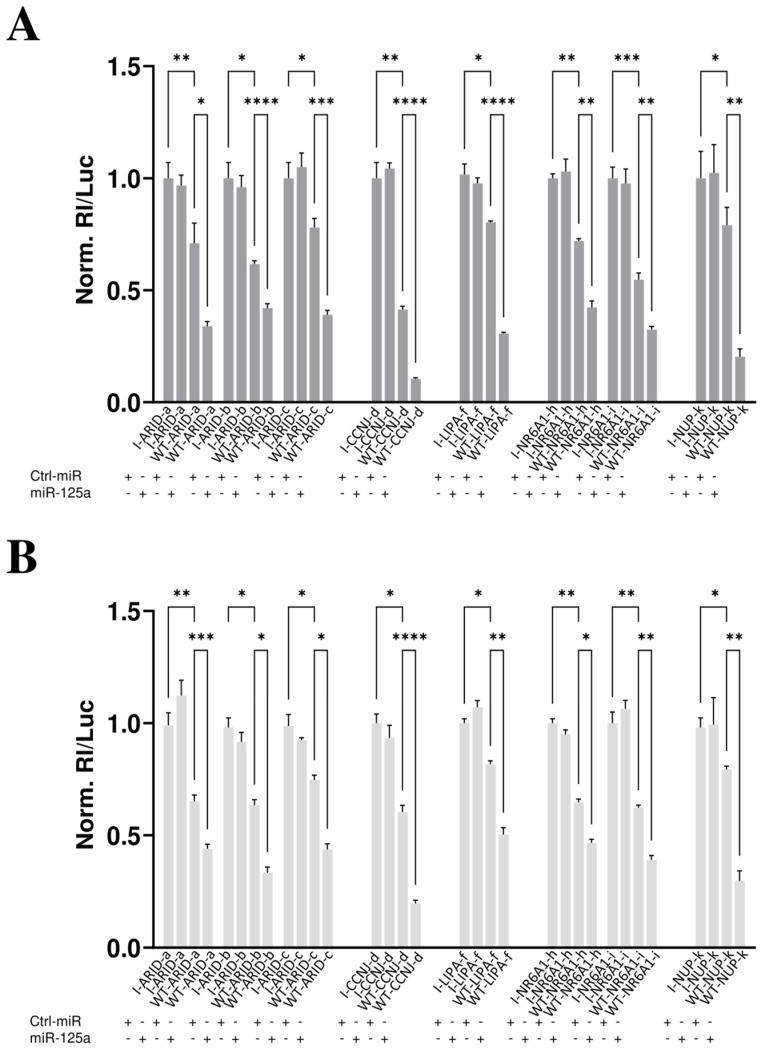
Validation of interaction between potential target sites and miR-125a-5p. HuH-7 (**A**) and HepG2 (**B**) cells were transfected with the luciferase-based reporter constructs containing the wild-type target sequence of miRNA (WT-target name-letter corresponding to the pairing of Figure 4) or the control plasmids carrying the inverted target sequence (code as above but indicated as I-), along with 50 nM of miR-125a mimic (+) or negative control, Ctrl-miR (+). After 48 h, luciferase activities were recorded. The *Renilla reniformis* luciferase activity (Rl) was normalized to the *Photinus pyralis* firefly luciferase activity (Luc), whose coding sequence is carried by the same vectors. The values are reported as fold mean + SD relative to Rl/Luc recorded for control transfections of I constructs with Ctrl-miRNA set to 1. Data are the mean +SD of two independent experiments, each with three data sets. *p*-values at Student’s *t*-test were * *p* < 0.05, ** *p* < 0.01, *** *p* < 0.001, or **** *p* < 0.0001.

The potential target sites were tested by reporter vectors transfected in HCC cells in combination with miR-125a mimic or its negative control molecule, Ctrl-miRNA, that did not match any target sequence. In brief, all the potential miR-125a binding sites were singularly cloned downstream the coding sequence of *Renilla reniformis* luciferase carried by the vector (code WT). The same approach was used to produce all the control reporter vectors carrying the inverted binding sequences (code I). The rationale of the assay is based on the fact that, after transfection, the vectors will produce the chimeric mRNAs, i.e., luciferase coding sequence with the potential WT binding sites in the 3′ UTR, and if they are bound by the miRNA, luciferase production will be inhibited, and a reduced bioluminescence will be registered in comparison to the controls represented by the I control vectors transfections. Figure 5 reports results of luciferase assays that validated the binding of miR-125a to the targets. In fact, in comparison to the controls (I-constructs co-transfected along with Ctrl-miR or miR-125a mimic), the luciferase was resultantly inhibited after transfection of WT reporter vectors along with Ctrl-miRNA, possibly as the consequence of the activity of the endogenous miR-125a, ranging from approximately 20% to 60% of luciferase reduction. An additional and significant reduction, leading to a total luciferase inhibition ranging from approximately 60% to more than 85%, was observed when the WT reporter vectors were transfected along with miR-125a mimic, thus confirming the specific inhibitory effect of miR-125a, due to its interaction with target sequences. In particular, all three putative target sites for ARID3A were validated (Figure 4, from a to c sites), two out of the predicted three for NR6A1 (Figure 4, h and i sites), one out of two for NUP210 (Figure 4, k site), and the others for CCNJ (Figure 4, d site) and LIPA (Figure 4, f site). Overall, the data demonstrated that the down-regulation of the above mentioned transcripts, observed by RNAseq experiments, was due to the physical interaction between the miRNA and the mRNAs, and they can be considered novel targets of miR-125a.

**Figure 6:**
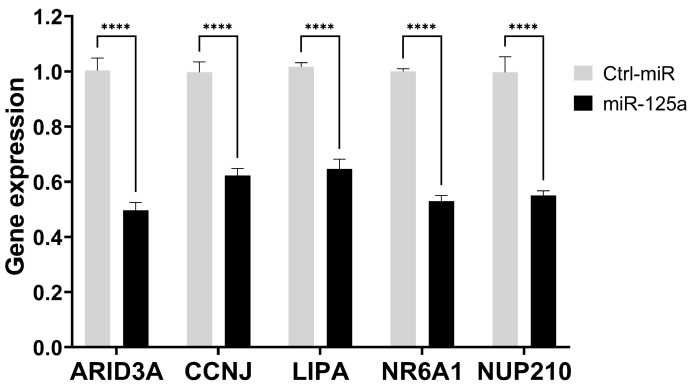
miR-125a silencing toward its oncogenic targets. The expression level of the indicated miR-125a targets was evaluated using RT-qPCR on RNA purified from HuH-7 cells transfected with miR-125a mimic or Ctrl-miR. The expression values are reported as fold mean (2−ΔΔCt) relative to control. Data are the mean + SD of replicate experiments. **** *p* < 0.0001 at Student’s *t*-test.

Finally, the down-regulation of ARID3A, CCNJ, LIPA, NR6A1, and NUP210 expression, as consequence of miR-125a increased level, was further evaluated by qPCR, giving values ranging from 50% to 35% of inhibition in comparison to the control (Figure 6), consistently with data from RNAseq experiments (Supplementary S1).

## 4. Discussion

HCC is one of the most prevalent cancers worldwide, with a high mortality rate due to diagnostic delay and limited effective treatment options. A minority of patients (10–20%) showing early stage HCC can be treated with surgical resection and liver transplantation. However, most HCC patients are diagnosed at advanced stages, and those options cannot be applied. Trans-arterial chemoembolization (TACE) and systemic chemotherapies represent the standard therapy for advanced HCC patients; in particular, multi-tyrosine kinase inhibitors such as Sorafenib, Lenvatinib, and Regorafenib have been approved for advanced HCC treatment, although they can only extend survival for three months [40,41]. Given the poor survival and high recurrence rate of HCC, it is a priority to study the underlying molecular mechanisms of HCC onset and progression for developing new diagnostic tools and innovative therapeutic strategies. Recently, many functional studies have highlighted the pivotal role of a plethora of ncRNAs, especially microRNAs, in hepatocarcinogenesis, also unveiling their potential as therapeutic targets [6]. In addition, both tissue and circulating miRNAs signatures have been increasingly associated with HCC diagnostics, since they can be significantly correlated to increased HCC development, advanced stages, prognosis, and overall patients’ survival [42,43,44].

As mentioned in the Introduction, different studies demonstrated the oncosuppressive role of miR-125a. The evidence of its ability to limit the tumor growth in vivo is particularly meaningful [15,16]. MiR-125a is a well-studied microRNA, since it is a homologous of lin-4, the first discovered miRNA in *C. elegans*, with various targets already characterized, such as Lin-28, a conserved target from nematodes to humans [24,45]. However, specific targets and molecular mechanisms specifically relevant for the onset and progression of HCC are still poorly known. Here, to gain a genome-wide perspective of the whole targetome, an omic approach has been applied to two different HCC cell lines samples after increasing the level of miR-125a and observing many de-regulated genes (Figure 1 and Figure 2). Focusing on down-regulated genes, and filtering the results with bioinformatic predictive tools, different transcripts have been hypothesized to be direct targets of the miRNA and subjected to experimental validation by reporter assays (Figure 4 and Figure 5). Some potential binding sites failed the validation tests, showing that target prediction is still challenging. Intriguingly, all the TargetScan predicted binding sites defined as 7mer-A1, independently of their evolutionary conservation, did not work in our experimental system. However, novel interesting targets have been identified, further validated by qPCR (Figure 6), as detailed below.

ARID3A, member of AT-rich interaction domain (ARID) family, is a transcriptional factor that has been already implicated in HCC, up-regulated in tumor samples, partly due to DNA demethylation at its gene locus, and related to a poor outcome [46,47,48,49]. An interesting mechanism of its oncogenic effect is based on the interaction between ARID3A and CEP131, that together transcriptionally activate KDM3A expression, which in turn demethylates H3K9me2 (H3 lysine 9 dimethylation) in the promoter regions of embryonic stem cell-like genes signature, leading to their up-regulation and thus facilitating liver cancer malignancy [46]. ARID3A activities of inhibiting cell differentiation, promoting cell proliferation, and increasing cell survival potential have been implicated in some developmental pathways, and reported as oncogenic in different other cancer types, with the binding site “a” of Figure 4 validated in esophageal squamous cell carcinoma [50,51,52,53,54]. Here, all three predicted binding sites have been validated, indicating ARID3A as the best target of the miRNA. CCNJ, the cyclin J, controls the G2/M cell cycle transition and has been implicated as oncogene in different tumors, such as breast and gastric cancer [30,55], bladder cancer [56], and non-small cell lung cancer [57]. Here, it has been validated as a novel target of miR-125a, indicating a possible role in HCC. LIPA, lysosomal acid lipase A, is a critical enzyme involved in cholesterol metabolism, since it hydrolyzes cholesteryl esters and triglycerides for producing free fatty acids and cholesterol [58]. Interestingly, LIPA was recently discovered to be implicated in cancer as a molecular target of a powerful therapeutic small molecule (ERX-41) for triple-negative breast cancer and potentially other solid tumors: ERX-41 induces endoplasmic reticulum stress (ERS) resulting in tumor cell death, dependently on LIPA protein-folding activities in the ER and independently of its enzymatic function, resulting in the accumulation of unfolded proteins, causing ERS [59]. This novel LIPA-targeted therapy has been demonstrated as potentially effective also for ovarian cancer [60], where LIPA is up-regulated, as well as in renal carcinoma [61]. LIPA validation as a target of miR-125a suggests that the cited studies and therapies are worth to be explored also in HCC. NR6A1, Nuclear receptor subfamily 6, group A, member 1, is an orphan member of the nuclear receptor superfamily, and a transcriptional repressor essential for development [62]. It has been implicated in various cancer types such as gastric cancer, prostate cancer, and papillary thyroid carcinoma [63,64,65]. Importantly, NR6A1 is reported as an oncogene in HCC: it is up-regulated in HCC patients and associated with a poor prognosis; it promotes cell proliferation, migration, invasiveness, and tumor growth in vitro and in vivo, most likely through the cell cycle, mTOR, WNT, and ERBB signaling pathways [66,67,68,69,70]. Intriguingly, miR-125a-3p, produced from the same pre-miRNA of the -5p, also targets NR6A1 in pancreatic cancer cells [71], particularly linking NR6A1 to the miRNA, with two out of three predicted sites validated as binding sites for miR-125a-5p in this study. NUP210 is a nucleoporin whose primary function is the formation of pores to regulate the exchange between nucleoplasm and cytoplasm; however, it has been recently demonstrated to have a role in cancer. Specifically, in liver cancer, it is up-regulated and an oncogene, working as a key target and partner of SMARCB1, a common subunit of SWI/SNF chromatin remodeling complexes [72]. Moreover, NUP210 has been defined as a metastasis susceptibility factor, since it is responsive to mechanical signals of the extracellular microenvironment and promotes lung metastasis in mouse models of breast cancer through alteration of the mechanical response, focal adhesion, and cell migration [73]. Other works confirmed its oncogenic role in various cancer types, such as colorectal cancer [74], breast cancer [75], and acute myeloid leukemia [76]. Here, its validation as a target of miR-125a indicates a possible new molecular mechanism contributing to the oncosuppressive activity of the miRNA.

## 5. Conclusions

This study identified and validated novel miR-125a targets in HCC; interestingly, they have been reported as oncogenes in different tumor types, suggesting a general relevance of the miRNA in carcinogenesis through common pathways. Intriguingly, some targets, such as NR6A1 and ARID3A validated in this study, and Lin-28 found in other studies and here confirmed as down-regulated in both cell lines after miR-125a boosting, have a role also in developmental pathways, thus falling into a conceptual class of genes termed ‘‘oncofetal’’, i.e., highly expressed in the embryo, inactive in most adult tissues, and re-expressed/up-regulated/reactivated in tumors [77,78,79]. We speculated that some RNA networks relying on the miRNA are involved in cancer biology, mirroring the development, wherein they, as gatekeeper molecules, can regulate the transition between pluripotency and commitment to differentiation in development; in cancer they are reactivated regulating cell proliferation and tumor progression. It is also relevant that many genes have been found to be up-regulated as a consequence of miR-125a increased level (Figure 2). In this regard, it should be noted that two of the novel targets here discovered are transcriptional factors (NR6A1 and ARID3A), whose affected expression can impact the expression of many other downstream genes.

The molecular mechanisms and RNA interactions investigated in this work could be exploited in the future for identifying novel biomarkers and therapeutic targets in HCC.

## Data Availability

Sequencing data are available in the ArrayExpress database repository (https://www.ebi.ac.uk/arrayexpress/, accessed on 16 December 2024) with the following accession number: E-MTAB-14731.

## Supplementary Materials

The following supporting information can be downloaded at: https://www.mdpi.com/article/10.3390/biom15010144/s1, Supplementary S1: Excel file consisting of 6 sheets, reporting differential expressed (DE) genes in HepG2 or HuH-7 cells with increased level of miR-125a in comparison to control (DE_HepG2 and DE_HuH-7, respectively), validated miR-125a targets as reported in miRTarBase (miRTarBase), predicted targetome by both TargetScan and miRDB (Predicted targetome), merging lists of down-regulated DE-genes and predicted targetome in HepG2 and HuH-7 (Merging HepG2 and Merging HuH-7, respectively). Supplementary S2: Excel file consisting of 2 sheets, reporting full list of KEGG pathways enrichment analysis of DE-genes from HepG2 and HuH-7 cells (KEGG_HepG2 and KEGG_HuH-7, respectively).
